# High frequency of simultaneous presence of ESBL and carbapenemase producers among nosocomial coliform isolates in Faisalabad, Pakistan

**DOI:** 10.12669/pjms.37.1.3192

**Published:** 2021

**Authors:** Sofia Irfan, Aysha Azhar, Asad Bashir, Salman Ahmed, Abdul Haque

**Affiliations:** 1 Sofia Irfan, B.Sc. (Hons.). Department of Health Biotechnology, Akhuwat FIRST, Faisalabad, Pakistan; 2 Aysha Azhar, PhD. Postgraduate Research Laboratory, The University of Faisalabad, Faisalabad, Pakistan; 3 Asad Bashir, MPhil. Department of Health Biotechnology, Akhuwat FIRST, Faisalabad, Pakistan; 4 Salman Ahmed, B.Sc. (Hons.). Department of Health Biotechnology, Akhuwat FIRST, Faisalabad, Pakistan; 5 Abdul Haque, PhD. Department of Health Biotechnology, Akhuwat FIRST, Faisalabad, Pakistan

**Keywords:** *E.coli*, *K. pneumoniae*, Extended Spectrum beta-lactamase (ESBL), Carbapenemase

## Abstract

**Objectives:**

The objective of the current study was to find prevalence of relevant ESBL and carbapenemase producing genes in nosocomial *E. coli* and *K. pneumoniae* isolates and to check phenotypic susceptibility of all ESBL positive isolates to carbapenems.

**Methods:**

Forty ESBL producing clinical isolates of *Escherichia coli* (n=33) and *Klebsiella pneumoniae* (n=7) were examined for the presence of β-lactamase genes (CTX-M, CTX-M-1, 2, 3, 4 and TEM). Carbapenem resistance was checked phenotypically and by presence of *blaNDM-1* gene.

**Results:**

Nine (27%) were positive for CTX-M genes, and 10 (30%) for TEM among *E. coli* isolates. Importantly, six isolates showed co-existence of CTX-M and TEM genes. In *K. pneumoniae*, two (28%) isolates were positive for CTX-M and one (14%) for TEM genes. Eight (24%) *E. coli* and one (14%) *K. pneumoniae* isolates were positive for CTX-M-1. Respective figures for CTX-M-4 were three (10%) and one (14%). CTX-M-2 and CTX-M-3 groups were not represented. Twenty (50%) isolates were resistant to both imipenem and meropenem out of which only four isolates expressed *blaNDM-1* gene.

**Conclusions:**

The significant presence of both ESBL and carbapenemase producers and co-existence of ESBL and carbapenemases in the same isolates is worrisome.

**Abbreviations:**
ESBL: Extended spectrum β-lactamase.
MBL: Metallo-betlactamase.
PCR: Polymerase chain reaction.

## INTRODUCTION

Enterobacteriaceae are one of the main causes of bacterial infections in the world. In this family, *Escherichia coli* and *Klebsiella* spp. are the most prevalent among causes of nosocomial infections. A broad spectrum of clinical infections are caused by these pathogens in both immunocmpetent and immunocompromised patients. They are also among major causes of nosocomial infections.^1^

One of the most important therapeutic choices for treating infections in both humans and animals is β-lactam antibiotics. The increase in bacterial resistance to these antibiotics over the past twenty years is due to selective pressure caused by use and misuse of these antibiotics. The most relevant mechanism of resistance is the production of β-lactamases, which hydrolyze the β--lactam ring of these antibiotics.^2^ Point mutations produce variants which produce the so-called extended spectrum β-lactamases (ESBLs) responsible for extensive drug resistance.

After detection of the first pathogen that was found expressing ESBL in Germany in 1983,^3^ it was defined as β -lactamases capable of hydrolysing third-generation cephalosporins and aztreonam, but inhibited by clavulanic acid.

Resistance to β -lactams mediated by (ESBL) is especially relevant in *Enterobacteriaceae*.^4^ In recent years, these enzymes have acquired a high capability of dissemination, especially in clinical environments. Beside the initially described TEM and SHV ESBLs, more recently CTX-M, OXA, and PER β-lactamases have been reported.^5^

Infections caused by ESBL producing *E. coli* and *Klebsiella spp* are often treated by carbapenems. Among carbapenems, imipenem (IPM) and meropenem (MEM) have been most extensively used. However, resistance towards these drugs has started to emerge due to the production of carbapenamases that degrade all β-lactam antimicrobials.^6^

One of the major modes of action of these enzymes is to promote membrane impermeability which usually results in hydrolyzation of all penicillins, cephalosporins, and carbapenems. Mutations or alterations in porin channels may also cause membrane impermeability resulting in porin non-functionality or may be due to complete loss of the OmpF and/or OmpC porin proteins. Active drug efflux may also play a role.^7^

The emergence of carbapenem resistance has aggravated the situations as carbapenems are considered the last line of defense against these pathogens. It is the need of the hour to create awareness and device better control measures to halt emergence of drug resistance among *Enterobacteriaceae* throughout the world.^8^

The main objective of the current study was to explore the phenotypic spectrum of ESBL and carbapenemase producing *E. coli* and *K. pneumoniae* among isolates from a local nosocomial source using disc diffusion method and to identify relevant genes in an effort to help control measures. This study has specifically addressed ESBL and carbapenemase related resistance towards β-lactam antimicrobials which were once considered the effective antimicrobials with good safety profiles. Resistance mechanisms for other antimicrobial groups were not included in this study. Another limitation is that it is related only to hospital infections.

## METHODS

### Collection of specimens

The bacterial isolates investigated in this study were taken from human blood, urine, sputum, pus, throat, and ear samples collected at Madinah Teaching Hospital (MTH) Laboratory, The University of Faisalabad, Faisalabad, Pakistan from September 2019 to February 2020. These samples were obtained from all age groups irrespective of the gender of the patients. After initial screening for ESBL productions by phenotypic test, the ESBL producing isolates were transported to Akhuwat Faisalabad Institute of Research Science and Technology (Akhuwat FIRST), Faisalabad, Pakistan.

### Biochemical Identification of E. coli and K. pneumonia

Biochemical identification of isolates was done by MR (Methyl Red) and VP (Voges-Proskauer) tests according to manufacturers’ instructions (Merck, Germany).

### Phenotypic detection of ESBL producers by double disc synergy method

An isolate showing enhanced zone of ≥5mm with cefotaxime combined with clavulanic acid as compared to a zone of antibiotic alone by disc diffusion method was phenotypically designated as ESBL producer.^9^

### Molecular confirmation of E. coli

PCR was used to confirm *E. coli* isolates by targeting a housekeeping gene *uidA* that encodes for ß-glucuronidase with primers *uidA*-F ATC ACC GTG GTG ACG CAT GTC GC and *uidA*-R CAC CAC GAT GCC ATG TTC ATC TGC.^10^ Each 100 µL reaction mixture comprised of 1x PCR buffer (50 mM KCl, 10mM Tris HCl; pH 8.3); 2.5 mM MgCl_2_; dNTP’s 0.2 mM each; 50 pmol of each primer; 5 U of recombinant Taq polymerase (Fermentas) and 20ng of DNA template. The thermal cycler conditions were: denaturation for five minutes at 94°C; 30 cycles of amplification at 94°C for one minute, 50°C for one minute and 72°C for one minute; and finally extension at 72°C for seven minutes. The PCR products were visualized by electrophoresis on 2% (w/v) agarose gel.

### Antimicrobial susceptibility testing

*E. coli* and *K. pneumoniae* isolates were tested for their susceptibility to carbapenems; imipenem (IPM), and meropenem (MEM) by disc diffusion method. The results were interpreted following the guidelines of National Committee for Clinical Laboratory Standards (NCCLS, 2015).^11^

### Molecular identification of antimicrobial drug resistance genes

PCR was used to detect *CTX-M, TEM, CTX-M-1, CTX-M-2, CTX-M-3, CTX-M-4* and *NDM-1* genes. The primer sequences and amplicon sizes are summarized in Table-I.

**Table-I:** Primer sequences for PCR of drug resistance genes in ESBL producers

Gene	Primers	Oligonucleotide Sequences	Amplicon Size (bp)
*blaCTX-M*	*CTX-M-F*	SCSATGTGCAGYACCAGTAA	585 21
	*CTX-M-R*	ACCAGAAYVAGCGGBGC	
*bla*TEM	*TEM-F*	ATGAGTATTCAACATTTCCG	867 22
	*TEM-R*	CTGACAGTTACCAATGCTTA	
*blaCTX-M-*	*CTX-M-1-F*	GACGATGTCACTGGCTGAGC	499 15
*1*	*CTX-M-1-R*	AGCCGCCGACGCTAATACA	
*blaCTX-M-2*	*CTX-M-2-F*	GCGACCTGGTTAACTACAATCC	351 15
	*CTX-M-2-R*	CGGTAGTATTGCCCTTAAGCC	
*blaCTX-M-3*	*CTX-M-3-F*	CGCTTTGCCATGTGCAGCACC	307 15
	*CTX-M-3-R*	GCTCAGTACGATCGAGCC	
*blaCTXM-4*	*CTX-M-4-F*	GCTGGAGAAAAGCAGCGGAG	474 15
	*CTX-M-4-R*	GTAAGCTGACGCAACGTCTG	
*blaNDM-1*	*NDM-1-F*	GGGCAGTCGCTTCCAACGGT	475 23
	*NDM-1-R*	GTAGTGCTCAGTGTCCGCAT	

DNA amplification was performed in a thermal cycler. Each 100 µL reaction mixture contained 10X PCR buffer (50 mM KCl, 10mMTris HCl; pH 8.3); 2.5 mM MgCl_2_; dNTPs 0.2 mM each; 50 pmol of each primer; 5 U of recombinant Taq polymerase (Fermentas) and 20ng of DNA template. PCR conditions for the *blaCTX-M, bla*TEM, *blaCTX-M-1, blaCTX-M-2, blaCTX-M-3* and *blaCTX-M-4* genes comprised an initial denaturation step for 5 minutes at 95°C, followed by 30 cycles at 95°C for 1minute, 45°C for 30 seconds and 72°C for one minute, with a final extension step at 72°C for 10 minutes. For *blaNDM-1*, the amplification cycle consisted of five minutes at 95°C, followed by 30 cycles at 94°C for 1 minute, 55°C for 1 minute and 72°C for one minute, with extension at 72°C for seven minutes. The PCR products were visualized by electrophoresis on 1.5% (w/v) agarose gel.

### Ethical Committee Approval

The study was approved by Research Committee (Ref: AKTFST/Misc/2020-104, Dated: 16-07-2020) of Akhuwat FIRST, Faisalabad, Pakistan.

## RESULTS

### Isolation and Biochemical Identification of Isolates

A total of 40 ESBL producing isolates showed rose pink, mucoid and non-mucoid colonies indicative of coliform bacteria. The biochemical tests by MR-VP identified 33 isolates as *E. coli* (82.5%) and 7 isolates as *K. pneumonia* (17.5%).

### PCR for Confirmation of E.coli Isolates

PCR confirmed the presence of *uidA* gene in 33 isolates.

### Antimicrobial Drug Susceptibility Testing by Disc Diffusion

Of 33 ESBL producing *E. coli*,18 (54.54%), and 16 (48.48%) were resistant to imipenem and meropenem respectively and 14/33 (42%) isolates were resistant to both of the carbapenems. Four out of seven (57.14%) *Klebsiella pneumoniae* were resistant to both Imipenem and Meropenem (Table-II).

**Table-II:** Antimicrobial susceptibility pattern of test isolates.

Antibiotics(10µg)	*Susceptibility Escherichia coli n=33*		*Susceptibility Klebsiella pneumoniae n=7*	
	Susceptible	Resistant	Susceptible	Resistant
Imipenem(IPM)	15	18	3	4
Meropenem (MEM)	17	16	3	4

### Molecular Identification of ESBL and Carbapenemase producing genes

Presence of *CTX-M* and *TEM* genes was observed in 13 (39%) isolates of *E. coli* and two (28.5%) isolates of *K. pneumoniae* (Table-III). Three (9%) isolates were positive for *CTX-M* gene alone, four (12%) for *TEM* gene alone and six (18%) showed amplification for both *CTX-M* and *TEM* genes in *E. coli*. So nine (27%) isolates were *CTX-M* and 10 (30%) were *TEM* positive among *E. coli* isolates. In case of *K. pneumoniae*, one isolate was positive for both *CTX-M* and *TEM* genes and one isolate showed amplification for *CTX-M* gene only. Overall, two (28%) isolates showed amplification for *CTX-M* gene.Primers were used to sub-classify four *CTX-M* genes. Only *CTX-M* *-1* and *CTX-M* *-4* were detected. No amplification was obtained for *CTX-M-2* and *CTX-M-3* genes (Table-III). When the *CTX-M* positive isolates were checked for *CTX-M* type 1, eight (24%) isolates of *E. col*i and one (14%) isolate of *K. pneumoniae* showed amplification. Three (9%) isolates of *E. coli* and one (14%) isolate of *K. pneumoniae* showed amplification for *CTX-M* type 4. All the ESBL producing isolates showing resistance to carbapenem were checked for the carbapenemase production. Four out of 20 (20%) isolates showed amplification for *NDM-1*gene (Table-III).

**Table-III:** Distribution of relevant genes in ESBL and CP producing Isolates.

Sl. No	Gene Name	E. coli (n=33)	K. pneumoniae(n=7)	Total (n=40)
1	*CTX-M*	9 (27%)	2 (28%)	11 (27.5%)
2	*TEM*	10 (30%)	1 (14%)	11 (27.5%)
3	*CTX-M-1*	8 (24%)	1 (14%)	9 (22.5)
4	*CTX-M-4*	3 (9%)	1 (14%)	4 (10%)
5	*NDM-1*	4/20 (20%)	0	4 (20%)

## DISCUSSION

ESBL producers are a great health threat especially in nosocomial settings due to their exceptional ability to hydrolyze the latest generations of β-lactam antibiotics. Their prevalence has increased in an alarming manner in recent years in both hospital and community settings.

The objective of the current study was to find the prevalence of relevant ESBL and carbapenemase producing genes in *E. coli* and *K. pneumoniae* isolates from a major hospital in Faisalabad and to check phenotypic susceptibility of all ESBL positive isolates to carbapenems (imipenem, meropenem). This was necessitated because phenotypic effect may be caused by multiple genes although it was not possible to include all reported genes in this study.

In this study, 40 ESBL producing *E. coli* (n=33) and *Klebsiella pneumoniae* (n=7) were included. According to our results, six (18%) isolates of *E. coli* harbored both *CTX-M* and *TEM* genes; three (9%) were positive for *CTX-M* gene alone and four (12%) for *TEM* gene alone. In total, nine (27%) isolates were positive for *CTX-M* gene and 10 (30%) isolates were positive for *TEM* gene. This finding is in agreement with the report of Coli and coworkers which showed presence of these genes in 35% isolates.^12^

Of the 7 ESBL producing *K. pneumoniae*, two (28%) isolates were positive for *CTX-M* gene and one (14%) isolate for *TEM* gene. This finding is in disagreement with a study in a hospital of Iran where 92% and 76% isolates were positive for *CTX-M* and *TEM* genes respectively.^13^

We further segregated the *CTX-M* positive isolates (*E. coli* 9/33 [27%] and *K. pneumoniae* 2/7 [28%]) to check the presence of various subclasses of *CTX-M* genes. Consensus primers, which recognize variant genes of *blaCTX-Ms* to date, were used. Only *CTX-M-1* and *CTX-M-4* genes were detected. *CTX-M-2* and *CTX-M-3* genes were not detected in any isolate.

Our finding of 24% isolates of *E. coli* testing positive for *CTX-M-1* β-lactamase gene is in strong agreement with a previous study in which 25% *E. coli* were reported to be *CTX-M-1* positive.^14^ Among our eight *E.coli* isolates, three were also carrying *CTX-M-4* gene.

We were able to detect one isolate of *K. pneumoniae* which was positive for both *CTX-M-1* and *CTX-M-4* genes. This is a unique finding as some other researchers were not able to detect *CTX-M-4* gene in any of *K. pneumoniae* isolates.^15^,^16^ The possible reasons might be the low level of resistance in community and nosocomial infections due to different geographical locations.

On the other hand, we were not able to detect *CTX-M-2* and *CTX-M-3* genes in any of our isolates. It is in contrast to the findings of some other researchers who were able to detect these genes in 5-10% isolates which might be from community settings while in our study all of the samples were obtained from tertiary healthcare hospital.^17^

Sixty percent of the 40 ESBL-producing isolates in this report were negative by PCR for *blaCTX-M*. This may be due to other/yet unexplored CTX-Ms. Another reason may be the presence of mutated *blaCTX-Ms*. It highlights the importance of the synergistic use of phenotypic methods and molecular methods for comprehensive understanding the resistance patterns and mechanisms.

We observed that approximately 50% of *E. coli* and 57% of *K. pneumoniae* isolates were resistant to both carbapenems, i.e., imipenem and meropenem phenotypically. This observation is in agreement with a report where resistance pattern was 60% and 70% against imipenem and meropenem respectively in *E. coli* isolates.^18^ However, our findings are not in agreement with a recent study in which only 30% isolates of *Klebsiella pneumoniae* were resistant to both imipenem and meropenem.^19^ The reason may be that we studied only isolates known to produce ESBLs.

According to a report on carbapenem-resistant *Enterobacteriaceae* isolates, *NDM-1* was detected in 75% (27/36) and 66% (10/15) *K. pneumoniae* and *E. coli* respectively.^20^ In our study, 50% ESBL producers (all *E. coli*) were carbapenemase producers and among them only four isolates (20%) were positive for *NDM-1*gene. No *K. pneumoniae* isolate was found positive. The reason may be that the carbapenem resistance may be due to some other recently discovered genes including OXA-48, KPC-3 and VIM-1 genes.^21^-^24^

## CONCLUSION

The co-existence of ESBL encoding genes (CTX-M-1 and CTX-M-4 group) in both *E. coli* and *K. pneumoniae* is indicative of rising threat posed by ESBL producing *E. coli* and *K. pneumoniae*. Moreover, the co-existence of ESBL and MBL in the same isolate indicated complete resistance towards all β-lactam antimicrobials especially the carbapenems which were considered the last wall of defense against these pathogens.

### Authors’ Contribution:

**SF:** Molecular Biology Experiments.

**AA:** Sample collection.

**AB:** Microbiology Experiments.

**SA:** Sample revival and verification.

**AH:** Concept and overall supervision.
